# Imaging appearance of a singular metastatic adenoid cystic carcinoma of the right kidney: A case report and literature review

**DOI:** 10.3892/ol.2014.2546

**Published:** 2014-09-17

**Authors:** DA-SHENG QIU, LI-YING XU, XIAO-YAN HU

**Affiliations:** 1 Department of Positron Emission Tomography/Computed Tomograpy, Hubei Cancer Hospital, Wuhan, Hubei 430079, P.R. China; 2 Department of Radiology, Zhongnan Hospital of Wuhan University, Wuhan, Hubei 430071, P.R. China

**Keywords:** metastasis, renal, ^18^F-fluorodeoxyglucose, positron emission tomography, computed tomography

## Abstract

Renal metastasis of a submandibular gland adenoid cystic carcinoma is clinically rare when it presents with an atypical imaging appearance of singular renal metastases. Whole-body positron emission tomography (PET)/computed tomography (CT) can determine whether the singular renal mass is benign or malignant and identify metastases in other parts of the body, particularly in uncommon sites. In the present case, the patient developed a rare partial metastasis to the right kidney three years after undergoing a surgery for submandibular gland adenoid cystic carcinoma. Based on the present case, whole-body PET/CT examination could provide an important basis for making treatment plans for singular renal metastases.

## Introduction

Adenoid cystic carcinoma is a rare, inert and invasive tumor. The occurrence rate only accounts for 1% of head and neck tumors and 22% of salivary gland tumors (1,2). The carcinoma commonly occurs in the minor salivary gland of the upper jaw, followed by the parotid and submandibular glands. Adenoid cystic carcinoma tends to invade the nerves and cause distant hematogenous metastasis (3), which is usually observed in the lungs (4), whereas renal metastasis is commonly observed in lung, breast and other cancers (5). Renal metastasis of a submandibular gland adenoid cystic carcinoma is rare. In addition, adenoid cystic carcinoma has a slow clinical progression and there is a long survival time associated with the tumor. In clinical practice, singular adenoid cystic carcinoma metastases are usually surgically removed. Pre-operative whole-body positron emission tomography (PET)/computed tomography (CT) is of great importance in the treatment of distant metastases of adenoid cystic carcinoma, particularly metastases to uncommon sites. The results of PET/CT examination are valuable when making treatment plans (6). The present study reports the case of a patient in whom a rare partial metastasis to the right kidney was detected by whole-body PET/CT following previous surgery for a submandibular gland adenoid carcinoma. Written informed consent was obtained from the patient.

## Case report

**Figure 1:**
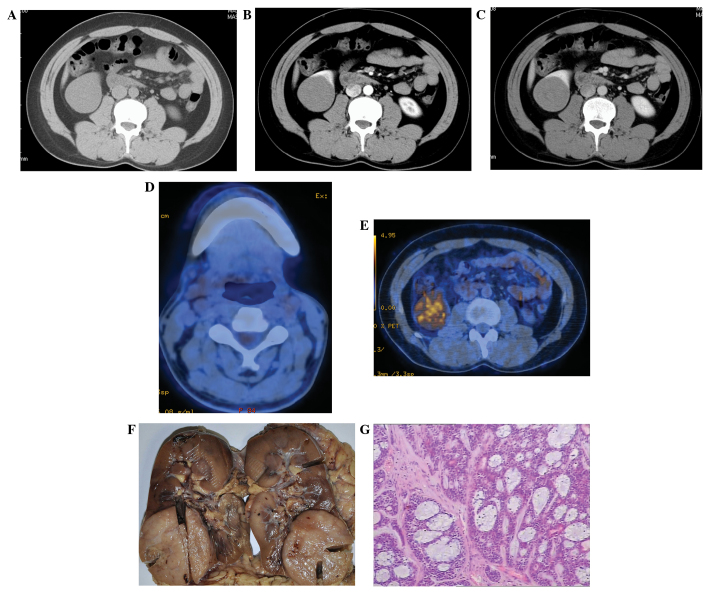
(A) Plain CT scan revealing a slightly lower-density mass, 5.5×5.0×5.0 cm in size, in the right kidney; the CT value was 36±5 HU. (B) Enhanced CT in arterial phase revealing a slightly enhanced mass, with a CT value of 48±5 HU. (C) Enhanced CT in the venous phase revealing an evidently enhanced lesion, with a CT value of 68±5 HU, which was high compared with the arterial phase.(D) ^18^F-fluorodeoxyglucose positron emission tomography-CT revealing left submandibular gland agenesis without local radioactive concentration. (E) Lump-shaped moderate radioactive concentration shadow in the lower right kidney (maximum standardized uptake value, 5.03). (F) The 5.0×6.0×5.0-cm hard mass in the lower right kidney, with a gray-white color and intact tumor capsule. (G) Hematoxylin-eosin staining of the adenoid cystic carcinoma in the right kidney (original magnification, ×40). CT, computed tomography.

A 26-year-old male, who had undergone post-operative radiotherapy for a left submandibular gland adenoid cystic carcinoma three years previously, presented to Hubei Cancer Hospital (Wuhan, China) the hospital due to an increasing level of pain in the right side of the waist, which had persisted for 10 days. No other discomfort, such as hematuria or edema, was reported. Tests for tumor markers were found to be negative. An enhanced CT scan revealed an enlarged right kidney with a slightly lower-density mass that was 5.5×5.0×5.0 cm in size in the lower right portion (Fig. 1A), with a CT value of 36±5 HU. The mass had clear edges and an even density. A slightly enhanced right kidney mass was observed in the arterial phase (Fig. 1B), with a CT value of 48±5 HU. The degree of mass enhancement in the venous phase was greater than that in the arterial phase (Fig. 1C), with a CT value of 68±5 HU. The patient was diagnosed with a right kidney tumor using CT. On PET/CT, post-operative changes were observed in the left submandibular gland (Fig. 1D). The imaging results for the kidneys were abnormal. A lump-shaped moderate radioactive concentration shadow was observed in the lower right kidney (Fig. 1E), with a maximum standardized uptake value of 5.03. No evident abnormal radioactive concentration shadow was observed in any other part of the body. A right radical nephrectomy was performed at the Hubei Cancer Hospital.

The right kidney was 15.0×15.0×10.0 cm in size at the time of the surgery. A 5.0×6.0×5.0-cm hard mass could be observed in the lower kidney (Fig. 1F), with a gray-white color and an intact tumor capsule. However, the previously the normal capsule of lower kidney had been invaded by the tumor. The renal fascia was smooth and no significantly enlarged lymph nodes were observed in the renal hilum. The finding from the examination of an intraoperative rapid frozen section indicated an epithelial malignant tumor. Based on the post-operative hematoxylin-eosin staining, which was compatible with a right kidney adenoid cystic carcinoma (Fig. 1G), combined with the patient’s medical history, a renal metastasis of the submandibular gland adenoid cystic carcinoma was considered to be the diagnosis. The mass was resected and the patient received no further treatment.

## Discussion

Based on autopsy reports, the incidence rate of renal metastasis is 3–15%, and this mainly originates from lung (19.8–23.3%), mammary gland (12.3%), stomach (11.1–15.1%) and colon cancers (22.2%) and melanoma (14.8%) (5,7). Renal metastasis is caused by hematogenous metastases of primary tumors, usually multiple or multifocal. The patient in the present case only presented with a singular metastasis in the lower right kidney, which made the mass difficult to clinically diagnose. Submandibular gland adenoid cystic carcinoma is rare and to the best of our knowledge, has never been reported in the literature. Metastatic renal carcinoma does not have clear symptoms or exhibit hematuria in the majority of cases. In the present case, the patient exhibited no clear clinical symptoms, with the exception of an increasing level of pain in the right side of the waist. Microscopic hematuria has a low incidence rate in metastatic renal carcinoma. This is mainly due to the renal metastasis being located in the vascular plexus cortex adjacent to the glomeruli, therefore, it relatively infrequently invades the urothelium (8). The patient in the present case did not have evident urinary symptoms. Only the increasing pain level in the waist was observed, caused by the large tumor size stimulating the renal fascia.

Diverse CT features of renal metastasis have been reported in the literature, mainly presenting as solid or cystic masses and hemorrhagic or diffuse lesions (8). The patient in the present case possessed a singular solid mass with a slightly lower density, as revealed by a CT scan. The mass had clear edges. The lesion was large and shared an indistinct boundary with the kidney capsule. Angiography of the renal metastasis revealed a hypovascular tumor. The majority of renal metastases are slightly enhanced, according to the literature (9). The present patient had a slightly enhanced lower right kidney mass in the arterial phase of CT enhancement (mean CT value, 36±5 HU; CT value in the arterial phase, 48±5 HU). The degree of mass enhancement in the venous phase was greater compared with the arterial phase (mean CT value, 68±5 HU). The enhancement pattern was different to that of classic primary renal carcinoma. The hemodynamic features of primary renal carcinoma exhibit a fast-in, fast-out enhancement pattern, with cancellation of the contrast agent in the venous phase (10). Therefore, the analysis of the enhancement features of this lesion provided the basis for the clinical diagnosis in the present study.

The ^18^F-fluorodeoxyglucose (^18^F-FDG) PET features of metastatic renal carcinoma are relatively infrequently reported in the literature (11). The patient in the present case mainly exhibited high FDG intake, indicating a significantly increased glucose intake by the tumor. Thus, diagnosis of a malignant lesion was considered. However, it remained difficult to determine whether the tumor was a primary or metastatic renal carcinoma. Metastatic renal carcinoma is usually a manifestation of malignant tumor systemic metastasis, normally with metastases in other parts of the body. However, in the present case, the only lesion identified was in the right kidney, and this was found through systemic PET/CT examination. Eventually a surgical resection treatment plan was clinically developed. In conclusion, careful analysis of multiple imaging findings combined with a review of the medical history of a patient could increase the accuracy of imaging diagnosis. PET/CT findings provide an essential basis for making treatment plans for renal metastasis.
